# Masking recurrent contaminants in reference sequences improves specificity of clinical metagenomic sequencing

**DOI:** 10.1128/jcm.00397-26

**Published:** 2026-07-20

**Authors:** Judith Bergada-Pijuan, Ian Pichler, Maryam Zaheri, Verena Kufner, Michael Huber

**Affiliations:** 1 Institute of Medical Virology, University of Zurich 27217, Zürich, Switzerland; Vanderbilt University Medical Center, Nashville, Tennessee, USA

**Keywords:** bioinformatics pipeline, masking, database curation, virus diagnostics, database contamination, metagenomic sequencing, clinical metagenomics

## Abstract

Viral metagenomic next-generation sequencing (mNGS) is a powerful approach for pathogen detection in clinical diagnostics; however, accurate virus identification depends critically on the quality of reference databases. Diagnostic specificity is frequently compromised by erroneous viral classifications, which occur when host- or reagent-derived sequences align to non-viral contaminant regions (such as ribosomal RNA, vector contamination like cytomegalovirus enhancers, and sequencing adapters) embedded within the viral reference sequences. To address this, we present VirMask, a novel computational strategy to systematically identify and mask recurrent contaminant regions within the viral reference sequences. In a first step, VirMask aligns simulated human reads against a viral database and masks host-derived regions. Second, it identifies and masks persistent contaminant regions based on their high prevalence across independent metagenomic data sets. Finally, VirMask employs alignment-based similarity searches to detect and mask homologous regions across multiple reference sequences, thereby reducing noise in mNGS outputs and improving diagnostic specificity. Using data from clinical mNGS runs, we demonstrate that VirMask usefully reduces artefactual detections without impacting true pathogen identification. Specifically, reads erroneously assigned to human viruses decreased by up to 30%, and those assigned to non-human viruses and bacteriophages by more than 99%. Furthermore, validation with a standardized international quality control panel confirmed 100% preservation of true-positive detections, while reducing false-positive human virus calls by up to 89% and overall erroneous assignments by 40%–94%. These results underscore the necessity of rigorous viral database curation and offer a reproducible framework for enhancing diagnostic confidence in mNGS-based clinical virology.

**IMPORTANCE:**

The importance of this study lies in addressing a critical bottleneck in clinical metagenomic next-generation sequencing (mNGS): the presence of systematic false-positive viral detections caused by contaminant regions within reference databases. While mNGS is a powerful, unbiased tool for pathogen discovery, its diagnostic reliability is often compromised by “kitome-derived” sequences that align to non-viral segments embedded in viral reference genomes. By introducing VirMask, this research provides a reproducible framework to systematically identify and mask these recurrent artifacts without sacrificing the sensitivity required to detect true pathogens. This targeted refinement of viral databases significantly reduces “noise” in diagnostic outputs, ensuring that clinicians can interpret metagenomic data with higher confidence and avoid misidentifying persistent laboratory contaminants as clinically significant infections.

## INTRODUCTION

Metagenomic next-generation sequencing (mNGS) has revolutionized infectious disease diagnostics by enabling the unbiased detection of microbial nucleic acids directly from clinical samples (1–3). Unlike targeted PCR-based methods, mNGS does not require prior assumptions about the causative agent, allowing for the identification of a broad spectrum of pathogens, including novel or unexpected viruses, in a single assay. This approach has proven especially valuable in diagnosing infections of unknown etiology, investigating outbreaks, and detecting pathogens in immunocompromised individuals (4–6).

Despite its broad utility, the reliability of mNGS for viral detection depends heavily on the quality and curation of the underlying reference database used for taxonomic classification. Most pipelines and stand-alone tools (e.g., our in-house VirMet [https://github.com/medvir/VirMet], along with widely used approaches such as SURPI [Sequence-based Ultra-Rapid Pathogen Identification] [7], metaMix [8], DAMIAN [9], Vipie [10], MetaMIC [11], Kraken 2 [12], and Centrifuge [13]), rely on publicly available comprehensive databases, including the NCBI viral RefSeq or GenBank repositories (14, 15). While these repositories provide extensive viral diversity, their reference sequences may also contain regions of non-viral origin, called here “contaminant regions,” to which sequencing reads of human, microbial, or environmental origin can align (16, 17).

Host depletion and computational filtering steps are routinely applied in diagnostics to remove host-derived and background reads (e.g., VirMet incorporates several depletion stages targeting human, bacterial, fungal, and bovine DNA). Still, several classes of contaminant nucleic acids can persist in metagenomic data. First, rRNA sequences originating from the host, environment, and contaminant nucleic acids introduced by laboratory reagents (“kitome”) may remain if they are not specifically depleted during sample preparation (18–20). Second, residual sequencing adapter and transposase sequences originating from library preparation kits may persist in mNGS reads when trimming is omitted or insufficient and might consequently be incorporated into viral reference sequences (21). Finally, the laboratory workflow frequently introduces contaminants of viral origin, including widespread environmental plasmid vectors harboring viral elements (e.g., cytomegalovirus [CMV] enhancers). These residual sequences (whether rRNA, technical adapters, or viral-derived elements) can subsequently align to viral reference sequences that inadvertently contain such contaminants (17, 22). Clinical mNGS data frequently contain these artifacts, leading to recurrent, implausible detection of specific viral taxa across unrelated samples, resulting in systematic false-positive identifications.

This problem is highly relevant in the diagnostic context, where even low-level detection of virus reads may be interpreted as clinically significant. Without robust controls or a curated database, clinicians and researchers risk drawing incorrect conclusions based on background noise or systematic artifacts. Previous studies have highlighted the extent of contaminant regions in public databases (23–25) and emphasized the need for improved database curation in metagenomics. However, relatively few studies have addressed this issue from a diagnostic perspective or proposed structured approaches to decrease recurrent false positives in viral mNGS workflows.

To mitigate these issues, clinical mNGS facilities and pipeline developers frequently rely on semi-curated databases. This curation often involves manually whitelisting well-annotated genomes, blacklisting known problematic accession numbers, or employing heuristic filtering to remove host-homologous regions. For example, the developers of established pipelines like SURPI (7) have explicitly emphasized the necessity of constructing accurate, well-annotated reference databases to mitigate false-positive hits. Additionally, broader reviews in the field highlight ongoing efforts to build curated databases combining annotated sequences from multiple sources (1, 3). However, manual curation is labor-intensive and struggles to keep pace with unpredictable, newly emerging laboratory artifacts.

While automated tools such as DeconSeq (16), Conterminator (23), and the method by Lu and Salzberg (25) effectively remove known genomic contaminants (e.g., host or bacterial sequences), they are fundamentally designed for targeted decontamination; hence, the contaminant taxa must be defined *a priori*. DeconSeq specifically requires pre-defined “retain” and “remove” databases to dictate which sequences are kept or discarded (16). While excellent for expected contaminants, clinical diagnostics suffer heavily from unpredictable artifacts. Consequently, building a “remove” database for unknown laboratory artifacts is often unfeasible. Similarly, Conterminator is designed for cross-kingdom contamination but cannot handle intra-kingdom viral dynamics because it excludes viral genomes by design (23). Hence, when used to identify contaminant sequences that are frequently embedded within the viral genomes (e.g., Illumina primers), Conterminator fails to find these recurrent contaminants. Finally, the method by Lu and Salzberg uses Kraken to clean reference databases (25). This method, which was originally developed for detecting contaminant regions in eukaryotic pathogen genomes, mirrors the routine filtering steps applied in mNGS diagnostics to remove host and background reads (such as those employed by our in-house VirMet pipeline). Therefore, even if adapted to clean viral genomes, it would suffer from the same drawback: the detection of persistent, initially unexpected contaminants would remain a challenge. Ultimately, state-of-the-art tools are not fully tailored to the specific challenges of viral diagnostics and offer limited strategies to prevent spurious viral misclassifications, as they overlook non-viral regions embedded within viral reference sequences, as well as residual laboratory-derived artifacts. This underscores the need for an untargeted, virus-focused approach capable of systematically improving the reliability of virus assignments in diagnostic mNGS pipelines.

To address this gap, we developed VirMask, a novel computational strategy to improve the specificity of virus detection by masking contaminant regions within the viral reference database. While VirMask is entirely database-agnostic and can be applied to any custom reference set, in this study, we utilized a comprehensive database derived from NCBI RefSeq (comprising 224,490 viral genomes). As a first step, our approach identifies and masks human-derived regions within virus reference sequences by aligning simulated human reads from the GRCh38 reference genome to the viral database and replacing any matching regions with ambiguous nucleotides. Next, it mines previous sequencing runs to identify recurrent contaminants, that is, short regions that get detected in a high proportion of samples or that consistently dominate the read counts whenever a given viral genome is detected, suggesting that the signal is overrepresented and may arise from technical artifacts or non-viral sources rather than genuine infections. These regions are then subjected to alignment-based similarity searches (using BLAST [26]) to identify homologs in the reference database, which are subsequently masked as well. This targeted masking reduces the likelihood that reads derived from non-viral origin or kitome contamination will align to viral reference sequences containing contaminant regions.

We applied VirMask to a large set of clinical metagenomic sequencing data processed through the VirMet pipeline, validated VirMask on Quality Control for Molecular Diagnostics (QCMD) samples, and demonstrated that our approach usefully mitigates the number of recurrent and unreliable viral classifications. Importantly, true-positive detections of clinically relevant viruses are preserved, underscoring the method’s utility in a diagnostic context. By improving the specificity of viral identification without sacrificing sensitivity, VirMask represents a practical and reproducible enhancement for mNGS-based diagnostics, contributing to more accurate interpretation of metagenomic data in clinical and public health laboratories.

## MATERIALS AND METHODS

### Viral reference database

Viral reference sequences were obtained from the NCBI RefSeq database (Taxonomy ID 10239, Viruses) (14) using the VirMet fetch command in July 2025 (https://github.com/medvir/VirMet). Only complete genomes or segments were included, and sequences derived from cellular organisms or human chromosomes were excluded. Accession numbers were retrieved via NCBI Entrez queries with Biopython (27), and sequences were downloaded using the NCBI data sets API v18.4.0 (28). Individual FASTA files were concatenated into a single-nucleotide reference database (*viral_database.fasta*), and duplicate sequences were removed using SeqKit v2.10.0 (29). To enable taxonomic classification in downstream BLAST-based analyses, the corresponding NCBI taxonomy databases were also downloaded and extracted.

**Fig 1:**
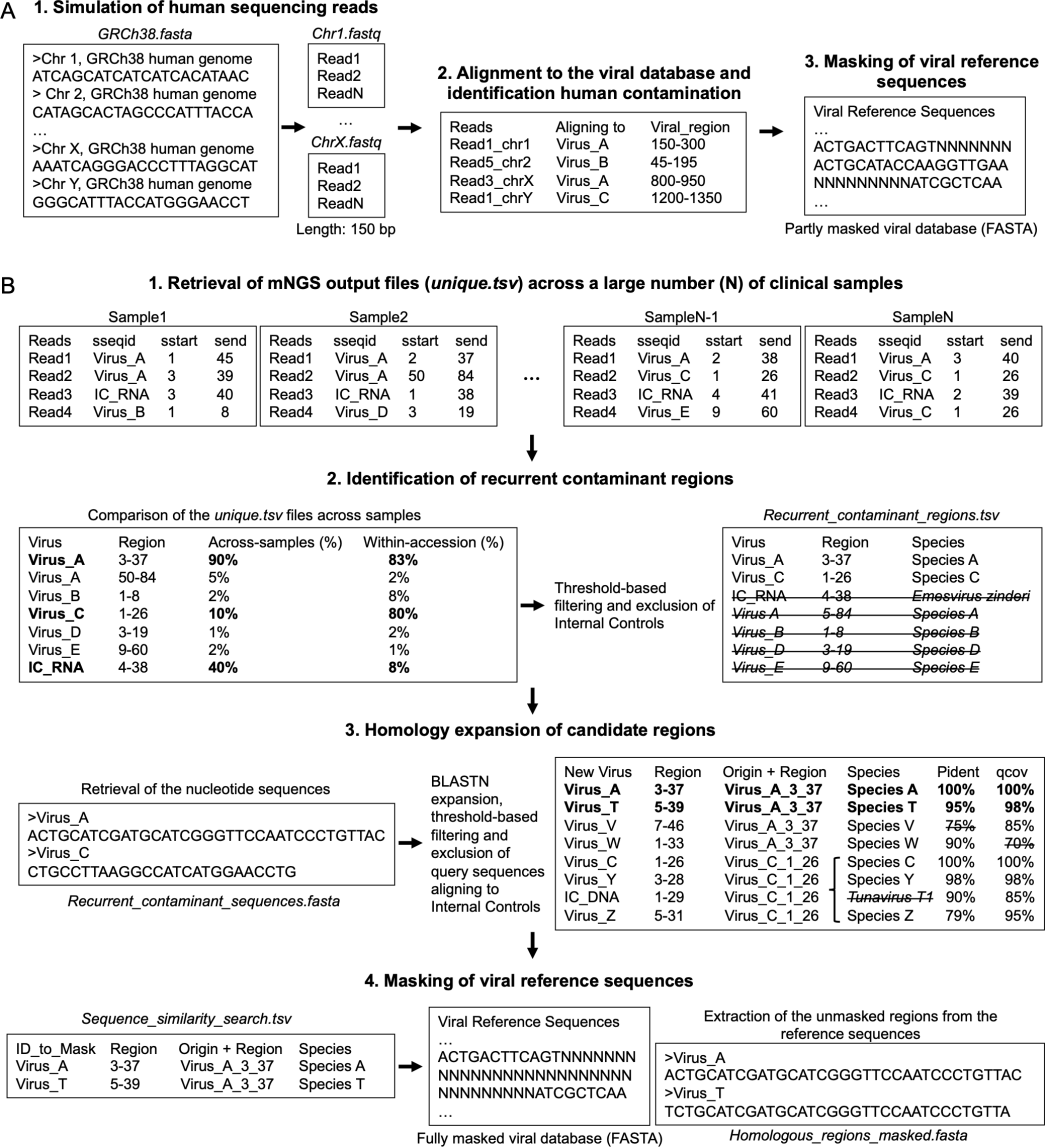
Workflow of the VirMask functionality. (**A**) Workflow of the *Human_masking.py* process. Initially, synthetic human sequencing reads are generated *in silico* based on the GRCh38 genome. These reads are subsequently mapped against the viral database, and the resulting matches from the viral references are masked by “N” substitution. (**B**) Workflow of the *Recurrent_masking.py* process. First, *unique.tsv* files from VirMet are gathered, and overlapping regions are compared across all samples. Next, regions that are repeatedly observed across samples or that are overrepresented in a specific viral isolate are classified as recurrent contaminants. A homology expansion step is later applied to all these candidate regions, leading to a final table of regions to mask. Internal controls or regions homologous to internal controls are excluded from the table. Finally, the contaminant regions are masked from the viral reference sequences by “N” substitution.

Aiming to minimize potential contamination from human sequencing reads in the downloaded viral reference database (referred to as “unmasked”), we implemented an initial masking approach combining simulated human read alignments with iterative database modification within VirMask (Fig. S1; *Human_masking.py*, calling [i] *Mask_human_regions.py* and [ii] *Summarise_human_regions.py*). For that, we generated simulated reads from each chromosome of the GRCh38 reference genome using ART v2.5.8 (Illumina, error-free, 150 bp reads) (30). These reads were aligned against the viral reference database with BWA-MEM v0.7.19 (31), and alignments were subsequently processed with samtools v1.21 (32) and bedtools v2.31.1 (33) to identify regions of the viral database with evidence of human read mapping. For each human chromosome, the corresponding regions of the reference sequences (*viral_database.fasta*) were masked, replacing aligned bases with ambiguous nucleotides (N). This procedure (visually shown in Fig. 1A) yielded a reference database (referred to as “partly masked”) where regions susceptible to spurious alignment of human reads (“human contaminants”) were removed, serving as the base for all the subsequent masking steps and VirMet analyses described below. Since the primary assembly of CRCh38 is highly curated and virtually free of accidental non-human DNA (34–36), the risk of masking non-human DNA using our approach is negligible.

### Clinical metagenomic sequencing data sets

To identify recurrent contaminants in the partly masked viral database, we evaluated a large collection of clinical mNGS sequencing runs generated at our institute. Only patient samples with ≥100,000 reads passing the quality filter were included; negative controls and undetermined reads (e.g., reads whose barcodes could not be recognized and assigned to a specific sample) were excluded. To ensure enough sample diversity and avoid masking undesired regions, we recommend at least 80 individual samples. For this study, we analyzed 86 independent clinical samples over a 6-month period (January–June 2025), with each sample processed through a DNA and RNA workflow, resulting in 172 FASTQ files.

Sample preparation was performed as described previously (2). Sequencing was performed in-house on the Illumina MiSeq platform, producing single-end reads of 150 nucleotides in length. All mNGS data sets were subjected to the standard VirMet workflow, which includes quality control, adapter trimming, and multiple read depletion stages targeting human, bacterial, fungal, and bovine reference genomes. Following these steps, VirMet maps the remaining reads to the viral reference database using BLAST (26), generating alignment summary files (*unique.tsv.gz*) that list viral accession numbers, species annotations, and genomic coordinates for all detected alignments. These *unique.tsv.gz* tables formed the basis for the contamination-mining procedure described below (further details in Fig. 1B). Prior to reporting the results for diagnosis, VirMet applies a final filter to the *unique.tsv.gz* files, retaining only BLAST hits with ≥75% identity and ≥75% query coverage (*orgs_list.tsv*). While this step removes some recurrent contaminants from the results, a substantial fraction persists, underscoring the importance of database curation and masking with tools such as VirMask.

### Identification of recurrent contaminant regions

Beyond human masking, VirMask features a second Python-based functionality (Fig. S1; *Recurrent_masking.py*, calling [i] *Extract_contaminants.py* and [ii] *Mask_contaminants.py*) to detect regions within viral reference sequences that were repeatedly observed across unrelated patient samples (“recurrent contaminants”). For each viral isolate (in particular, each NCBI accession number), overlapping alignment regions were compared across all samples. This information could be retrieved from the *unique.tsv.gz* files described above.

Candidate recurrent contaminant regions were defined if at least one of the following two criteria was met:

1. Across-sample frequency: regions identified in at least x% (default 20%) of all analyzed samples, regardless of their total abundance.
2. Within-accession dominance: regions representing at least x% (default 20%) of all reads aligned to a given viral isolate, with the additional requirement that the candidate region itself must be supported by at least 20 sequencing reads.

This dual-threshold strategy detects regions that consistently lead to spurious hits across samples as well as high-abundance artifacts specific to certain viral reference sequences. Thus, all regions that were repeatedly classified as viral in our mNGS data sets were treated as potential recurrent contaminants, along with regions that were overrepresented in a specific viral isolate.

Internal controls spiked into our mNGS samples (e.g., Emesvirus zinderi [MS2 bacteriophage] and Tunavirus T1 [Escherichia phage T1]) were excluded from the list of potential contaminants by string-matching the species names. The final set of recurrent contaminant regions was compiled into a comma-delimited file (*Recurrent_contaminant_regions.csv*, Data S1), representing coordinates within viral reference genomes that likely generate persistent false-positive assignments (for a visual representation of the whole process, see Fig. 1B).

### Expansion of candidate regions using sequence similarity searches

To account for homologous contaminant regions present in related viral reference sequences, we implemented a BLASTN-based expansion step into our pipeline (details in Fig. 1B). In particular, the nucleotide sequence from each candidate region from *Recurrent_contaminant_regions.csv* was extracted from the viral reference database (*Recurrent_contaminant_sequences.fasta*, Data S2). Only regions between 21 bp and 499 bp were retained for the BLASTN expansion process and compiled into a query FASTA file. These heuristic default limits were established to balance specificity and sensitivity, thereby minimizing unspecific masking from very short regions (which may match unrelated viral sequences by random chance) and excessive loss of true viral signal from very long ones (which risks masking entire viral genes).

The resulting query FASTA file was aligned against the complete viral reference database using BLASTN (NCBI BLAST + v2.16.0+) (26) with the parameters -task blastn and -max_target_seqs 1,000,000. The output was restricted to hits with >80% nucleotide identity and >80% query coverage. Any query sequence producing alignments to internal control viruses (MS2 or T1) was entirely excluded—that is, all of its alignments (including those to other viruses) were discarded to prevent the inclusion of control-derived fragments, which may be annotated under alternative names. For each viral reference sequence, overlapping BLAST hits were merged, ensuring comprehensive masking of homologous regions across all viral reference sequences (*Sequence_similarity_search.csv*, Data S3).

### Masking of viral reference sequences

The contaminant regions resulting from the previous step (*Sequence_similarity_search.csv*) were used to mask the viral reference database (Fig. 1B). Nucleotides of these regions were substituted with “N,” preserving the genome length. To avoid excessive masking that could compromise diagnostic sensitivity, the total length replaced per genome was limited to 5% of its size. If the cumulative length of the identified contaminant regions exceeded this 5% threshold, the masking process for that specific genome was aborted, and the reference sequence was retained in its original, unmasked state. Genomes shorter than 1 kb were exempt from this percentage limit, although VirMask issues a warning indicating that the threshold has been exceeded and reports the total number of nucleotides masked. This exception was justified because only viroids may be shorter than 1 kb, and they are not known to infect humans.

In addition to generating the masked viral database, our pipeline also extracts the recurrent contaminant regions from the unmodified reference sequences and stores them separately (*Homologous_regions_masked.fasta*, Data S4). This enables independent validation and use of the masked regions. All results presented in this study were obtained using these extracted regions.

### Code availability and reproducibility

VirMask is a modular workflow that consists of two main functions: *Human_masking.py*, which detects and masks human-derived regions from viral reference genomes, and *Recurrent_masking.py*, which identifies and masks recurrent contaminants within the viral database. Each of these functions can be used individually, or the whole pipeline can be run with a single command, *Virmask.sh* (Fig. S1).

VirMask is freely available at https://github.com/medvir/VirMask, and full documentation on how to use the pipeline is provided at https://medvir.github.io/VirMask/.

Importantly, VirMask allows users to modify multiple input parameters, providing high flexibility:

- Across sample frequency: default 20%.
- Within-accession dominance: default 20%.
- Minimum size of masked region: default 21 bp.
- Maximum size of masked region: default 499 bp.
- Maximum percentage of genome masked: default 5%.
- Spiked-in controls: default “Tunavirus T1, phage MS2.”
- mNGS runs to analyze (this is a required parameter, without defaults).

VirMask has been executed in a Linux environment (Ubuntu 24.04.3 LTS) and has been tested using Python 3.12.10. Further information about the required software dependencies can be found on the documentation webpage. We benchmarked the full *Virmask.sh* pipeline using the viral reference database described previously in the Methods section, which consists of 224,490 viral genomes comprising a total of 5,326,217,105 nucleotides, thus representing a file size of 5.34 GB. To mask human contamination, we used the GRCh38 reference genome, which requires about 800 MB. For the masking of recurrent contaminants, we used 172 FASTQ files (86 clinical samples) as described previously, with a total combined size of 92 GB. Running on a server equipped with 32 CPU cores and 256 GB of RAM, the complete end-to-end masking process required a computational runtime of approximately 4 days (1 day for the human contamination and 3 days for the recurrent contaminants). VirMask is expected to be maintained and used for database curation in routine diagnostics at our institute. Therefore, all changes and future version control updates will be visible in the mentioned GitHub repository.

## RESULTS

### Characterization of viral read profiles in clinical mNGS data sets

To identify recurrent contaminant regions within the partly masked database, we benefited from 86 independent clinical samples of diverse specimen types from 25 diagnostic mNGS runs, each processed through two workflows (DNA and RNA), and containing at least 100,000 reads passing quality control (Fig. S2A and B). In general, the RNA workflow produced more viral reads than the DNA workflow, with median counts of 6,200 and 800 reads per sample, respectively (Fig. S2C). Among these viral reads, human viruses predominated within the DNA libraries (median = 110 reads per sample), although their abundance was lower than that in the RNA libraries (median = 270 reads per sample). Within the RNA workflow, the Enterobacteria phage MS2 internal control accounted for the majority of viral reads (median = 3,200 reads per sample). Non-human viral reads were comparatively rare in both workflows (median = 2 reads per DNA sample and 30 reads per RNA sample; Fig. S2D).

### Dual 20% thresholds optimally define contaminant regions

VirMet analysis of the 172 mNGS files (86 samples and 2 workflows) using the partly masked database produced comprehensive tables of viral alignment intervals across all samples (*unique.tsv.gz* files), from which potential contaminant regions were inferred. To establish robust thresholds for our masking approach, we systematically varied *across-sample frequency* and *within-accession dominance* cutoffs in 10% increments while fixing the complementary parameter at 0%. Raising the *across-sample frequency* threshold reduced the number of masked regions, whereas raising the *within-accession dominance* threshold reduced their lengths (Fig. S3A and B).

To safeguard clinically relevant signal, Torque Teno virus (TTV), a ubiquitous commensal and our most frequently detected, PCR-confirmed virus, was used as a benchmark. Since TTV sequences were masked at a 10% cutoff for either parameter (Fig. S3C), dual 20% thresholds were selected as an optimal balance, minimizing the loss of genuine viral signal while still effectively capturing recurrent contaminant regions.

### Masking of the 182 identified contaminant regions affected 14 human viruses

**Fig 2:**
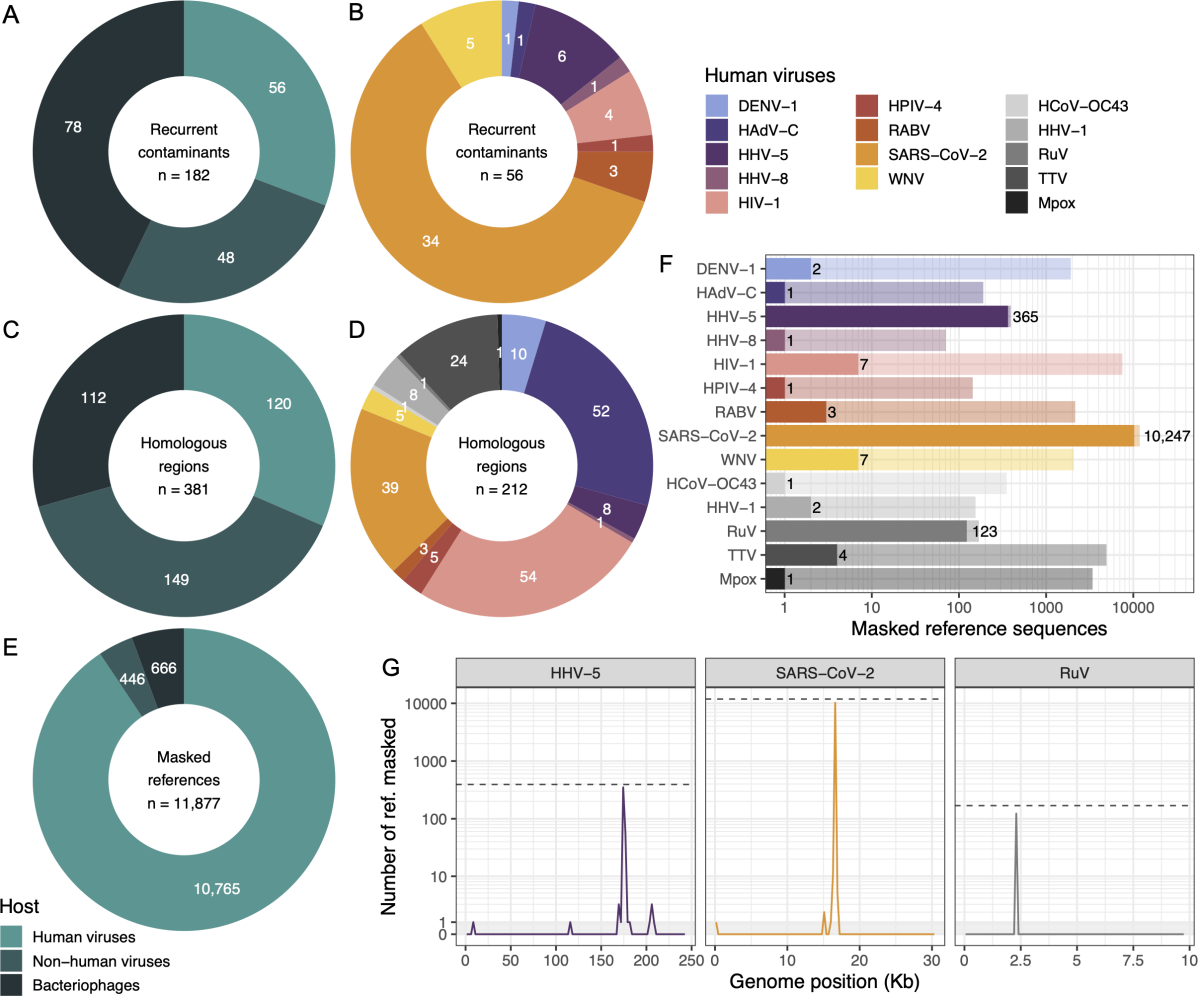
Masking of contaminant regions. Recurrent contaminants across (**A**) all viruses and (**B**) human viruses, annotated by their top BLAST hit. Homologous regions of recurrent contaminants across (**C**) all viruses and (**D**) human viruses upon sequence similarity search. (**E**) Reference sequences affected by masking contaminant regions. (**F**) Masked reference sequences per human virus (solid) out of all respective reference sequences in the NCBI database (transparent). (**G**) Loci of masked regions and number of affected reference sequences for HHV-5 (left), SARS-CoV-2 (center), and RuV (right). Dashed horizontal lines show the total reference sequence per virus in the database.

Applying the dual 20% thresholds, we identified 182 contaminant regions (Data S1 and S2), 56 of which were reported by VirMet as human viruses when using the partly masked database (Fig. 2A and B). To extend masking beyond individual reference sequences and to capture homologous regions present in related genomes, we performed sequence similarity searches across the entire viral database, as described in the Methods (Data S3). Following this homology-based expansion, several of the initial contaminants exhibited similarity to reference sequences from viruses infecting different hosts. In total, 112 of the 182 contaminants matched at least one bacteriophage, 149 matched at least one non-human virus, and 120 matched at least one human virus (Fig. 2C). Notably, many of the regions associated with human viruses aligned to multiple viral species rather than a single one, indicating that such recurrent regions are shared across unrelated taxa and likely represent persistent contaminants retained in the viral database. Consequently, these 120 regions aligned to 14 distinct human viral species (Fig. 2D). In total, nearly 12,000 reference genomes contained homologous regions that were masked (Data S4), 91% of which corresponded to human viruses (Fig. 2E).

To evaluate the breadth of this masking step, we first compared the number of masked reference genomes per human virus with the total number of reference genomes available for each viral species (Fig. 2F). This analysis revealed that the high proportion of masked human viruses was largely driven by the extensive representation of severe acute respiratory syndrome-related coronavirus 2 (SARS-CoV-2), which accounted for 95% of all masked viral reference sequences of human viruses. However, for most human viruses, only a small proportion of reference sequences was masked, indicating that contamination is confined to a few reference sequences and that masking these regions is unlikely to impede virus detection, since numerous unmasked reference sequences remain available. Because only a small subset of reference sequences contained these contaminants, it further suggests that such candidate regions represent true contamination regions rather than native viral sequences.

In contrast, contaminant regions were present across nearly all available genomes of these three viruses: human herpesvirus (HHV) 5, SARS-CoV-2, and *Rubella* virus (RuV) (Fig. 2F). Notably, these contaminants occupy short genomic intervals in each virus: 64 nt, 86 nt, and 29 nt, respectively (Fig. 2G; Data S3). Thus, masking them removes only a small fraction of the entire reference sequence (0.03%, 0.3%, and 0.3%, respectively), preserving most of the genome for reliable detection of these viruses. While a slight reduction in sensitivity might be suspected for HHV-5, SARS-CoV-2, and RuV infections with very low viral loads, quantitative analysis demonstrates that such a loss is highly improbable and can be neglected. Based on the exact binomial distribution, the *P*-values that one of three reads falls within the masked region of these viruses are 0.001, 0.007, and 0.037, respectively. If more than four reads align to these viruses, losing one would not result in any sensitivity loss because we consider three reads as a potential hit.

**Fig 3:**
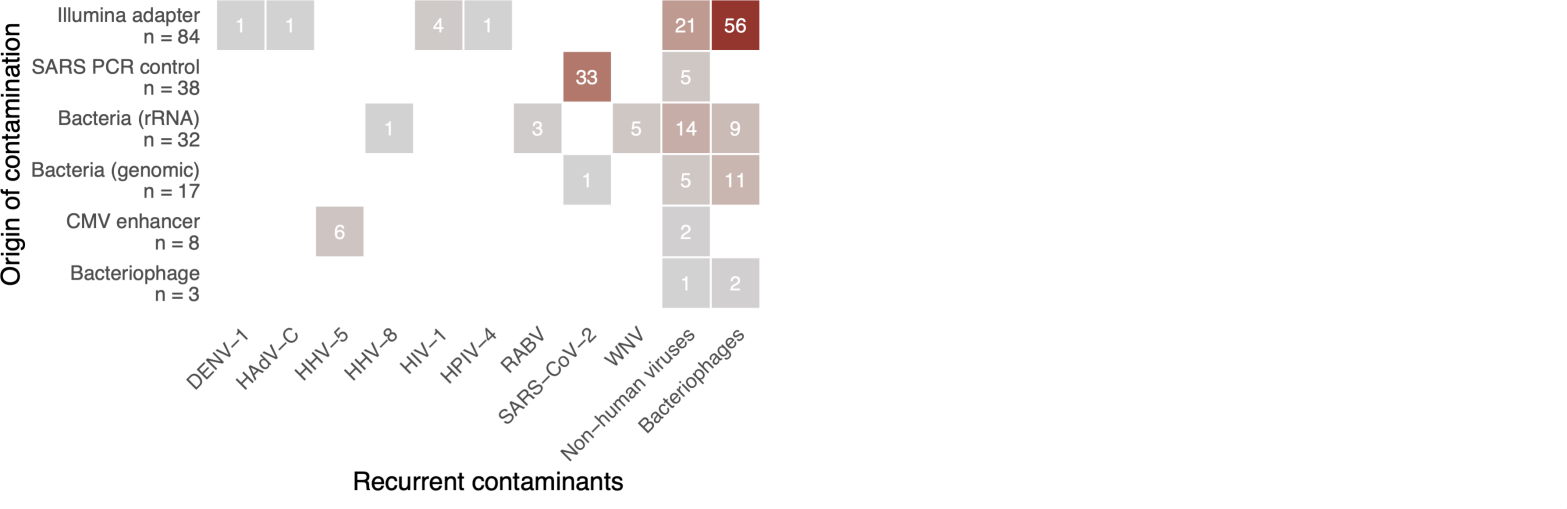
Origin of contamination. Recurrent contaminants (x-axis) with their respective origin of contamination (y-axis).

Initially, the recurrence of these homologous regions across nearly all HHV-5, RuV, and SARS-CoV-2 references suggested that they originated from authentic viral sequences. However, their frequent and consistent detection pointed instead to contaminant origins: the region in HHV-5 corresponded to the CMV enhancer, a common element in expression vectors used for enzyme production (i.e., a kitome contaminant); the region in RuV was homologous to the recurrent contaminant also detected in HHV-8 and could be traced back to rRNA; and the SARS-CoV-2-associated region was traced to a synthetic RNA control for a SARS-CoV-2 PCR targeting ORF1ab, indicating a laboratory-derived contaminant (Fig. 3). The exact NCBI accession numbers for all recurrent and homology-expanded masked regions are comprehensively listed in Data S1 and S3, respectively.

### Contaminant regions originate from six distinct sources

To determine the origins of all contaminant regions within the viral reference genomes, we performed BLASTN searches against the *Core nucleotide database* using quality thresholds equivalent to the initial human virus alignments (*Contamination_origin.csv*, Data S5). Given the high redundancy among masked regions, the 182 contaminant regions could be grouped into just six major categories of origin (Fig. 3). Nearly half corresponded to Illumina adapter sequences (which are not explicitly present or annotated as such in reference databases), followed by SARS-CoV-2 PCR control, bacterial genomic and rRNA sequences, the CMV enhancer sequence, and bacteriophages. Contaminant regions stemming from bacterial origin (genomic, rRNA, and bacteriophages) likely derive from the kitome, as bacteria are routinely used in enzyme production, analogous to the contribution of CMV enhancer sequences.

Beyond tracing the origins of contamination within the viral database and across clinical samples, we examined which viral species were introduced into the mNGS results as a consequence of these contaminants, as they pose a risk of false-positive reporting and clutter the results with spurious non-human viruses and bacteriophages (Fig. 3). Masking the Illumina adapters was particularly useful, as it prevented the erroneous detection of four human viruses in our analysis: dengue virus type 1 (DENV-1), human mastadenovirus C (HAdV-C), human immunodeficiency virus 1 (HIV-1), and human parainfluenza virus 4 (HPIV-4), along with 21 non-human viruses and 56 bacteriophages. Notably, besides HHV-5, the CMV enhancer sequence also introduced two non-human viruses into the analysis, although still within the Herpesviridae family: cyprinid herpesvirus 3 (KP343684.1) and alcelaphine gammaherpesvirus 1 (KX905134.1).

### Precise masking preserves clinically relevant viruses

**Fig 4:**
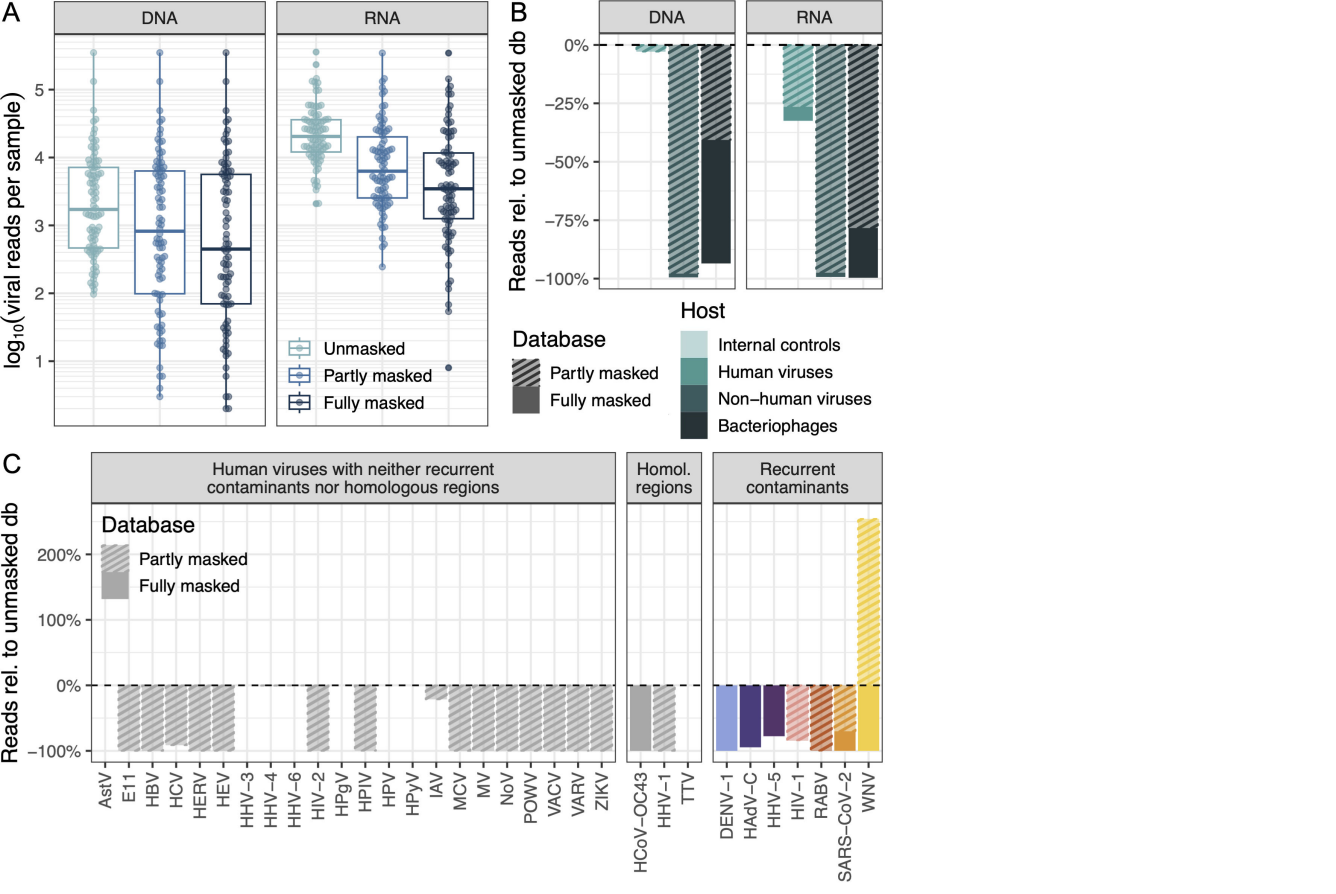
Effect of masking on viral reads. (**A**) Yield of viral reads per sample and workflow using unmasked, partly masked, and fully masked databases. Reported (**B**) viral reads and (**C**) human viral reads using the partly and fully masked database, relative to the unmasked database.

Masking human-derived regions and applying the dual 20% thresholds with VirMask to remove recurrent contaminants in the viral database markedly improved diagnostic interpretability. Across all samples, viral reads were substantially reduced from our mNGS results (Fig. 4A), with effects varying by virus category (Fig. S4A and B). The reductions in reads were most pronounced for non-human viruses, which decreased by 99.7% and 99.3% in the DNA and RNA workflows, respectively, and for bacteriophages, which decreased by 93.5% and 99.6% (Fig. 4B). As designed, internal controls were excluded from masking and, consequently, showed no effect (0.0%). Human viruses showed minimal reduction in the DNA workflow (<1%) but were substantially reduced in the RNA workflow (30.2%), likely reflecting the masking of rRNA-associated regions. To provide context for these read-level reductions, we investigated the absolute number of distinct viral taxa detected per sample before and after masking. Consistent with the decrease in reads, the fully masked database substantially dropped the number of human viruses, non-human viruses, and bacteriophages across all samples (Fig. S4C).

Following this overall reduction, a detailed analysis of the human viruses classified by VirMet revealed how both masking procedures reduced the number of detected taxa (Fig. 4C; Fig. S4D). Some species (astrovirus [AstV], HHV-3/4/6, human pegivirus [HPgV], human papillomavirus [HPV], human polyomavirus [HPyV], and TTV) remained unaffected, indicating genuine viral detections with minimal overlap between their reference genomes and human or contaminant regions. Other viruses (influenza A virus [IAV], hepatitis C virus [HCV], HHV-5, and HIV-1) were only partly affected: fewer samples tested positive for these viruses because reads mapped to unmasked regions only in a subset of samples, which likely reflects matches to alternative viral loci and is indicative of true-positive hits. In contrast, contaminants of several taxa were completely removed after human decontamination masking (*Human_masking.py*, the first step of VirMask), while others, including human coronavirus OC43 (HCoV-OC43), DENV-1, rabies virus (RABV), and West Nile virus (WNV), were eliminated after masking the recurrent contaminant regions in their reference genomes (*Recurrent_masking.py*, the second step of VirMask). The apparent increase in WNV-mapped reads following the initial human masking was caused by read reassignment, a well-known phenomenon in competitive metagenomics. When primary rRNA contaminants (such as a region of a *Saccharomyces cerevisiae* virus and a very short WNV region) were masked during the human decontamination step, the sequencing reads that were initially assigned to those regions realigned to a secondary, unmasked rRNA-homologous region within the WNV genome, creating a false-positive spike. However, this spike was resolved by the second masking step, which identified and removed the secondary target as well as all its homologous regions, confirming the artefactual nature of the alignment and validating the necessity of VirMask’s two-step architecture (see Fig. S5A and B for further details about this artifact).

### Validation on QCMD quality control samples

**Fig 5:**
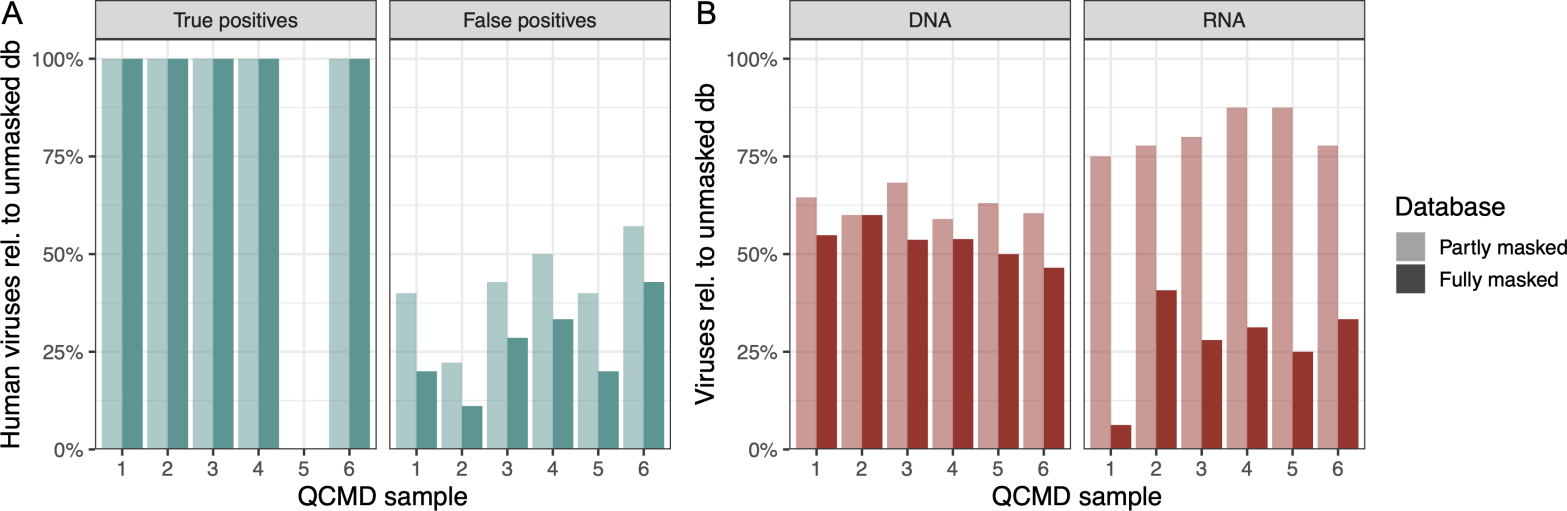
Validation on quality control samples. The results obtained with VirMet using the partly and fully masked databases, each expressed relative to the unmasked database baseline. (**A**) Ratios of true-positive and false-positive human virus detections (≥3 reads per virus) for the masked databases compared to the unmasked database. QCMD sample 5 contained no intended viral targets; accordingly, no true-positive values are shown in the true-positive panel. (**B**) Ratios of total detected virus species (≥3 reads per virus) for the masked databases compared to the unmasked database.

To assess whether masking preserves clinically relevant detections while reducing background signal in samples with known viral content, we analyzed a proficiency panel from the Quality Control for Molecular Diagnostics (QCMD) 2024 viral mNGS external quality assessment (EQA) pilot study. This panel, consisting of six samples, provides a curated quality control containing known DNA and RNA viruses at concentrations as low as 25 and 32,000 copies/mL, respectively. Sample 5 served as a negative control because it was not spiked with any viruses. We processed these samples using VirMet with the unmasked, partly masked, and fully masked reference databases generated by VirMask. All true-positive viruses remained detectable with the fully masked database, indicating that our masking approach with the dual 20% thresholds does not compromise diagnostic sensitivity. At the same time, the fully masked database consistently reduced the total number of false-positive human viruses detected per sample by at least 50%, and up to 89% (Fig. 5A). The remaining false positives predominantly reflected cross-sample contamination (index hopping) or laboratory carryover from routinely sequenced viruses. As these signals are typically amplicon-derived, they can be readily distinguished from true positives by coverage analysis. Importantly, parameter validation demonstrated that while increasing the masking thresholds above 20% did not negatively impact sensitivity (which remained at its maximum), it led to a progressive increase in false-positive calls, confirming 20% as the optimal cutoff (Fig. S6).

Consistent with earlier observations (Fig. 4B), the additional masking of recurrent contaminant regions (comparing to unmasked and partly masked databases) had minimal impact on DNA viruses but resulted in a pronounced reduction of false-positive RNA viruses (Fig. 5B). Using the QCMD panel, the combined effect of human and recurrent contaminant masking drastically simplified output files, decreasing the overall number of viral calls by 40%–53% for DNA samples and 59%–94% for RNA samples, thereby improving interpretability without losing clinically relevant information.

## DISCUSSION

Metagenomic virus detection is inherently prone to spurious signals arising from human, bacterial, and reagent-derived laboratory contaminants that can obscure true viral infections. By systematically masking recurrent contaminant regions in the viral reference database (and, as a preceding step, removing false human-derived sequences), VirMask substantially reduced false-positive hits while preserving the clinically meaningful virus reads. This approach not only resolved complex misalignments, such as reads driven by rRNA contamination that were falsely assigned to WNV, but also highlights the importance of careful reference curation in ensuring reliable interpretation of clinical metagenomic data.

To achieve effective masking of recurrent regions without removing genuine viral signal, we defined two thresholds: (i) across-sample frequency, representing the proportion of samples in which a contaminant region is observed, and (ii) within-accession dominance, representing the proportion of reads containing the region relative to all reads aligning to a given viral reference sequence. We selected 20% for both thresholds, which was the most stringent combination that did not mask TTV. TTV was chosen as a benchmark for true-positive signal because it is a commonly detected virus in blood (37), the primary material type tested in our laboratory (Fig. S2A). We note that these thresholds were optimized for our specific sample cohort and may not be universally applicable; VirMask therefore allows its users to define thresholds based on their own data set-specific characteristics.

Notably, all human virus contaminants detected at a 20% *within-accession dominance* threshold were also identified at a 70% threshold (Fig. S3C), highlighting their unlikely status as true-positive viral hits, since genuine viral reads are generally distributed evenly across the genome. Varying the *across-sample frequency* threshold further revealed that recurrent contaminant regions in DENV-1 and HAdV-C appeared in ≥ 80% of samples, making it highly improbable that these represent true positives. In contrast, any regions within WNV, HHV-5, and SARS-CoV-2 were only masked when the threshold was set below 50%, indicating that their contaminant regions were present in roughly half of the samples, likely reflecting detection exclusively in either the DNA or RNA workflow (Fig. S3C). Indeed, all three viruses were consistently detected only in RNA libraries. Reads assigned to WNV originated from bacterial rRNA (Fig. 3); reads assigned to HHV-5, although derived from a DNA element (the CMV enhancer), were also observed in RNA libraries, likely reflecting RNA workflow–specific reagent contamination, such as DNase treatment or reverse transcription. For SARS-CoV-2, the origin of the contaminant region was traced to a synthetic RNA control for a SARS-CoV-2 PCR targeting ORF1ab, indicating a laboratory-derived contaminant.

For three human viruses (HHV-5, SARS-CoV-2, and RuV), most reference sequences were masked, but only over very short genomic intervals (≤0.3%, Fig. 2F and G). Consequently, genuine viral reads can no longer align to these particular regions, but even at very low viral loads, the resulting loss of sensitivity is negligible. As ≥ 99.7% of the viral genome remains unmasked and available for alignment, a scenario where a positive call depends entirely on reads mapping to these masked intervals is statistically improbable (*P*-value < 0.05). Furthermore, isolated reads aligning exclusively to these intervals (which are linked to recurrent contaminants) would not constitute reliable evidence for a positive call.

VirMask’s strategy, combining the removal of contaminant human regions within viral references with the masking of recurrent contaminant regions, proved highly effective in eliminating non-human viruses and bacteriophages from reports and downstream analyses (Fig. 4B). This approach also substantially reduced spurious human virus detections in RNA libraries, lowering the number of reported taxa while maintaining a careful balance between true and false positives (Fig. 4C).

Validation on quality control samples demonstrated that systematic masking of contaminant regions could substantially reduce recurrent artefactual viral detections without compromising sensitivity for clinically relevant viruses (Fig. 5). The observed reduction in detected human virus species indicated that a considerable fraction of apparent viral diversity in mNGS data arises from persistent, non-biological signal rather than true infection. At the same time, the retention of all expected viruses across masking conditions supported the premise that such artifacts can be selectively removed without impairing diagnostic performance. While absolute “true-positive” counts are inherently difficult to define in heterogeneous clinical mNGS cohorts due to ubiquitous low-level noise (such as index hopping or laboratory carryover), this controlled validation confirms that VirMask safely isolates clinically meaningful signals. The residual false positives were largely attributable to these known sources of technical contamination and could be distinguished from true infections based on their genomic coverage profiles. Together, these findings suggest that database-level curation, as implemented in VirMask, addresses a major and previously underappreciated source of diagnostic noise in viral metagenomics.

While we demonstrate that VirMask improves the specificity of viral detection in Illumina-based clinical mNGS data generated within our laboratory, its performance has not yet been evaluated across independent data sets, sequencing platforms, or laboratory environments. Given known variability in reagent-derived background and workflow-specific biases, contaminant profiles are expected to be cohort- and laboratory-specific. VirMask is therefore intentionally designed as a data-adaptive framework, enabling users to define masking thresholds based on their own data set-specific background signals.

We note that this design implies a degree of local calibration rather than a fixed universal contaminant model. However, the extent to which learned contaminant profiles are transferable across sequencing technologies and laboratory environments has not yet been systematically evaluated. In particular, differences in library preparation, reagent composition, and sequencing technology may introduce distinct background signatures. Future work should therefore include systematic cross-platform and cross-laboratory validation to assess the robustness and transferability of VirMask-derived masking profiles.

Furthermore, while the present study focuses exclusively on curating a viral reference database, the algorithmic principles underlying VirMask are fundamentally organism-agnostic. The mathematical framework (identifying artefactual regions based on their suspiciously high across-sample frequencies and within-accession dominance percentages) relies purely on alignment metrics. Expanding this masking strategy to bacterial, fungal, or pan-microbial databases would represent a highly relevant next step toward fully agnostic diagnostic workflows. We acknowledge that curating bacterial databases would introduce distinct challenges, such as substantially larger genomic search spaces and the presence of highly conserved biological elements that must be protected from over-masking. Applying VirMask to these domains would require the provision of appropriate reference databases, prior metagenomic data, and careful recalibration of the masking thresholds. Nevertheless, the underlying software architecture remains highly adaptable, providing a conceptual foundation for future pan-pathogen database curation.

VirMask directly addresses a major source of false positives, a long-standing obstacle in metagenomic studies (38–40), by demonstrating that targeted masking meaningfully improves the reliability of virus detection. This successful validation underscores that careful database curation through masking is a viable and effective solution to a key field-wide challenge. The ability of VirMask to automatically mask any database, given adequate prior metagenomic data, establishes it as a versatile resource. Ultimately, this approach enhances the robustness of metagenomic data, enabling more definitive downstream analyses and interpretations.

## SUPPLEMENTAL MATERIAL

Data S1 jcm.00397-26-s0001.csv Set of recurrent contaminant regions representing coordinates (Start and End) within viral reference genomes (ACC) that likely generate persistent false-positive assignments (Species).

Data S2 jcm.00397-26-s0002.FASTA Extracted nucleotide sequence from each candidate region from recurrent contaminant regions.

Data S3 jcm.00397-26-s0003.csv List of BLASTN-based alignments resulting from the homology expansion step (where candidate contaminant regions were queried against the complete viral reference database), after filtering for >80% nucleotide identity and >80% query coverage, and excluding any queries matching MS2 or T1 control viruses. For each viral reference sequence (with sseqid, stitle, sscinames, and slen provided), overlapping hits have been merged to define contiguous homologous regions to be masked (sseqid, sstart, send).

Data S4 jcm.00397-26-s0004.FASTA Extracted nucleotide sequence from all homologous recurrent contaminant regions from sequence similarity search.

Data S5 jcm.00397-26-s0005.csv BLASTN-based taxonomic assignment of each contaminant region (with acc_start_end, length, and sequence provided) against the Core nucleotide database (NCBI), providing the origin of contamination (origin).

Fig. S1 to S6 jcm.00397-26-s0006.pdf Fig. S1: Overview of the VirMask workflow. Fig. S2: Overview of clinical samples. Fig. S3: Parameter selection to identify recurrent regions. Fig. S4: Effect of masking on viral reads (absolute numbers). Fig. S5: Effect of database masking on WNV read alignment. Fig. S6: Parameter validation on quality control samples.

ASM does not own the copyrights to Supplemental Material that may be linked to, or accessed through, an article. The authors have granted ASM a non-exclusive, world-wide license to publish the Supplemental Material files. Please contact the corresponding author directly for reuse.
