# Supplementary material for: Masking recurrent contaminants in reference sequences improves specificity of clinical metagenomic sequencing
Source: J Clin Microbiol. 2026 Jul 20;64(8):e00397-26. doi: 10.1128/jcm.00397-26 (PMC13463744; doi:10.1128/jcm.00397-26)
Supplement: Supplementary file 1 [file jcm.00397-26-s0006.pdf]

Supplementary Fig. 1: Overview of the VirMask workflow

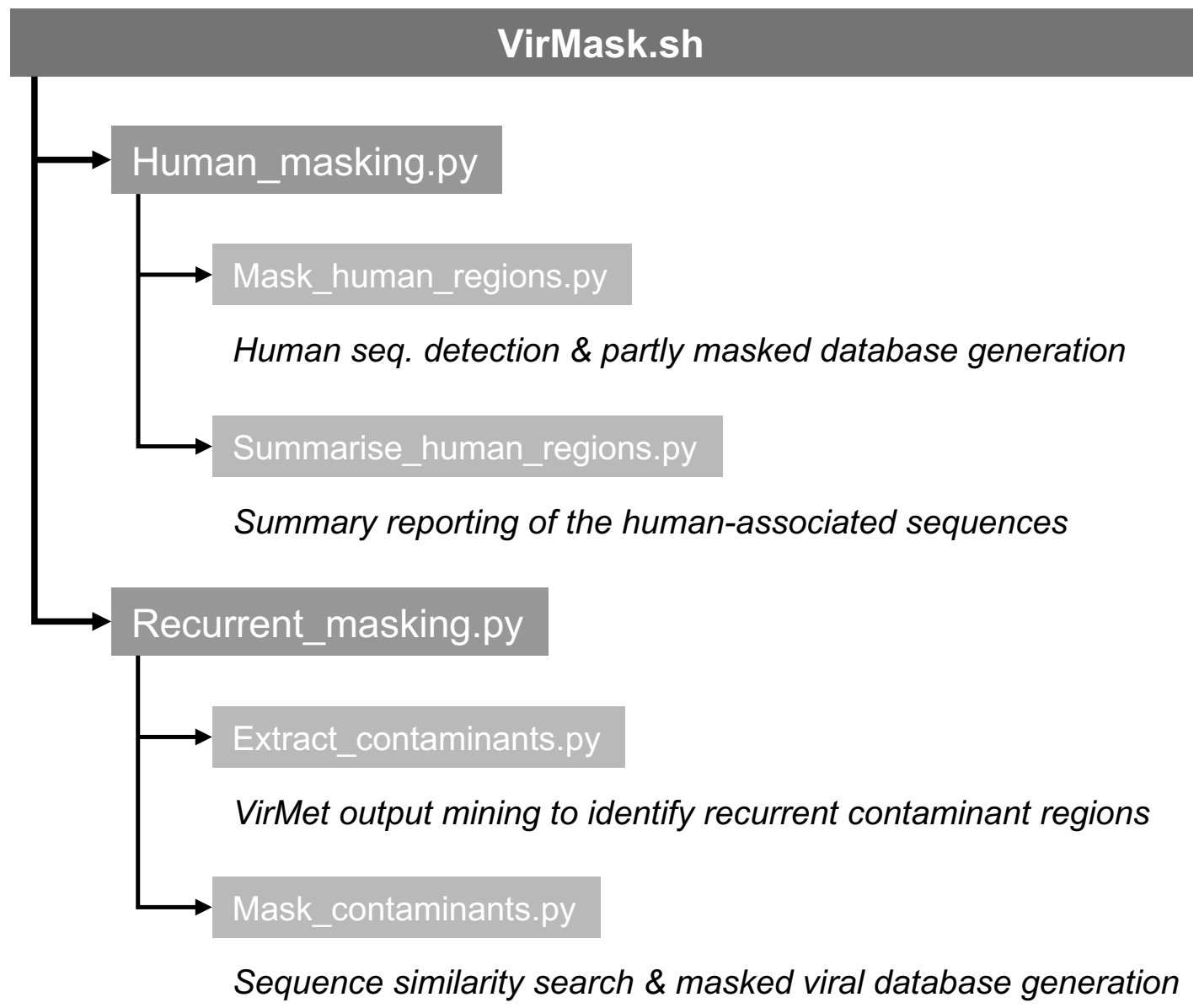

Schematic overview of the VirMask command pipeline. Each processing step can be executed independently, or the full analytical workflow can be run in an integrated manner as a single command**.**

Supplementary Fig. 2: Overview of clinical samples

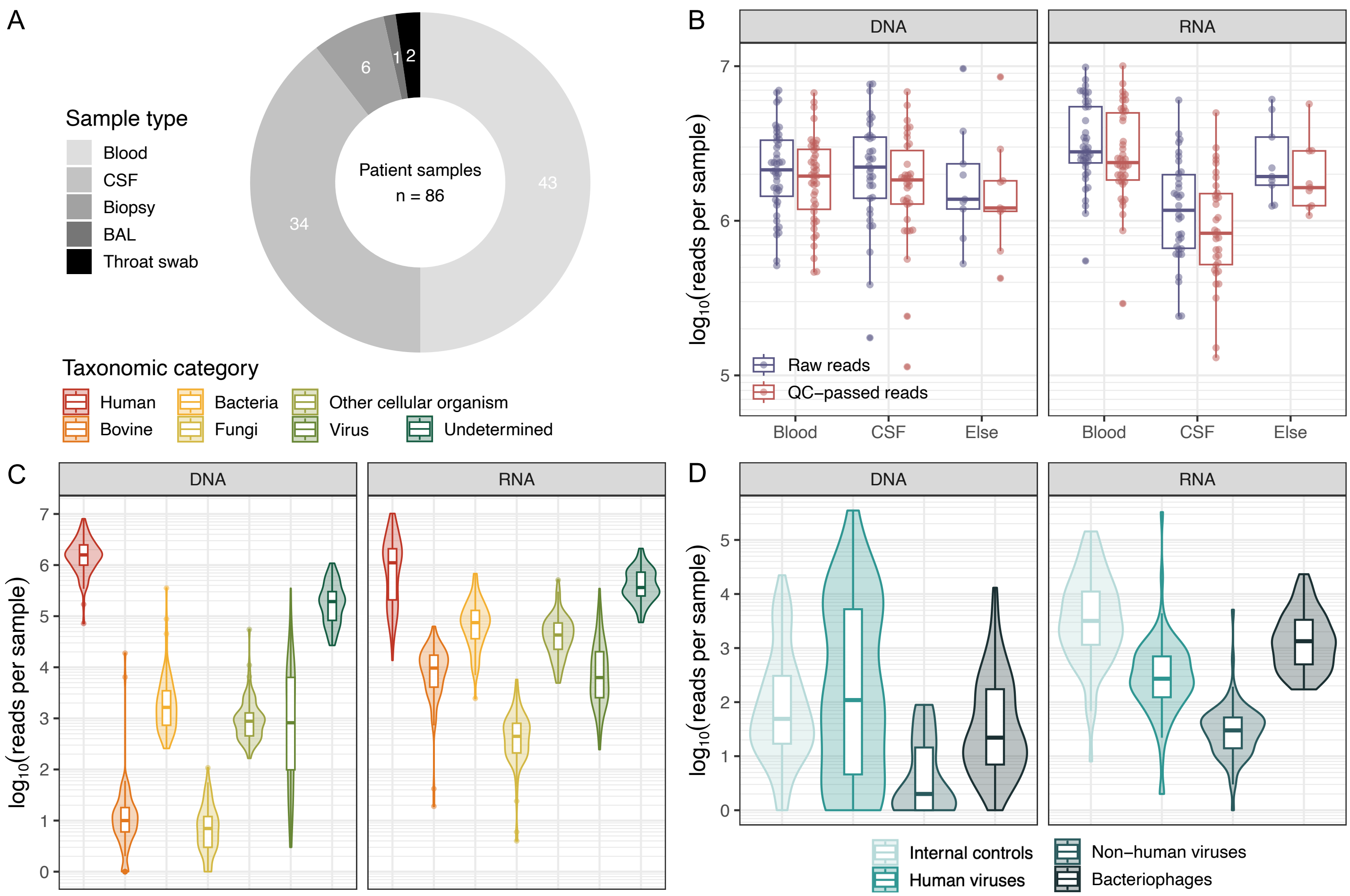

(**A**) Sample material types. (**B**) Read counts stratified by material type and workflow. (**C**) Taxonomic composition of reads per sample and workflow. (**D**) Viral composition of reads per sample and workflow.

Supplementary Fig. 3: Parameter selection to identify recurrent regions

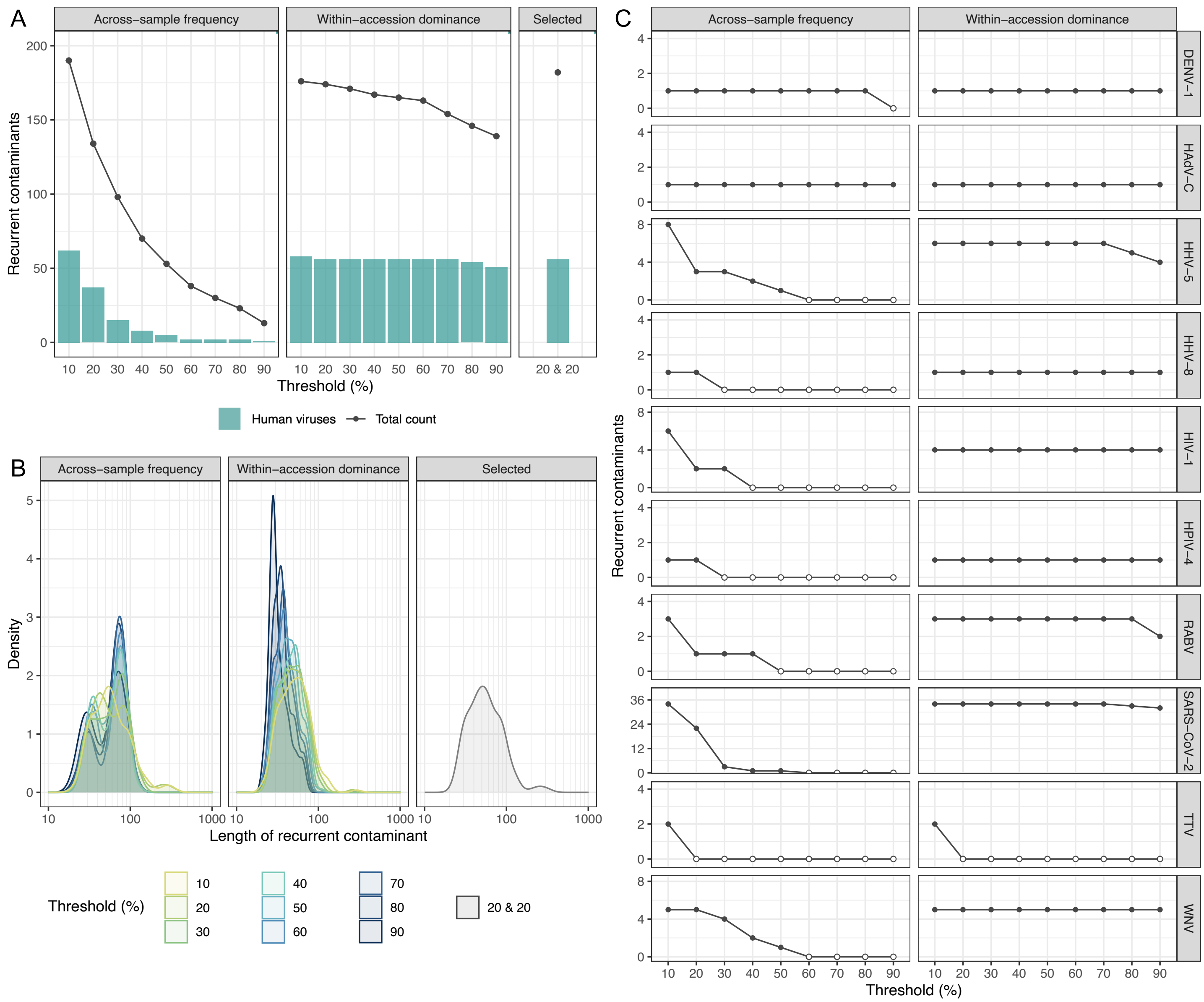

(**A**) Number of masked regions across varying thresholds of *across-sample frequency* (left) and *within-accession dominance* (centre), with the complementary parameter fixed at 0%, and for the selected dual 20% thresholds (right). Lines indicate total masked regions; bars denote subsets with top BLAST hits to human viruses. (**B**) Length distribution of masked regions under the same parameter settings. (**C**) Number of masked regions per human virus across thresholds of *across-sample frequency* (left) and *within-accession dominance* (right), with the complementary parameter fixed at 0%.

Supplementary Fig. 4: Effect of masking on viral reads (absolute numbers)

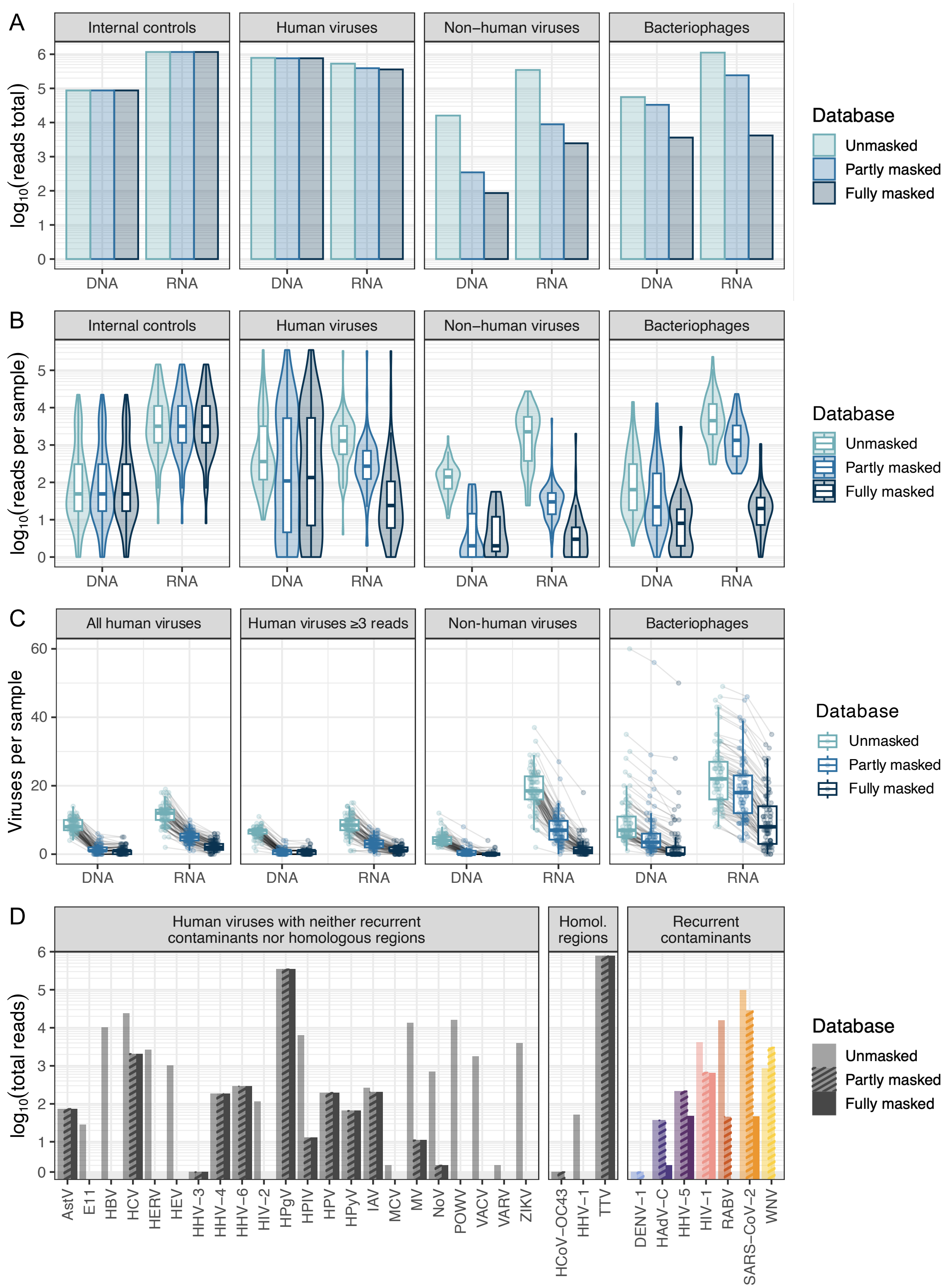

Reported (**A**) total and (**B**) per-sample viral reads per workflow obtained using the unmasked, partly masked, and fully masked databases. (**C**) Reported total human viral reads per workflow obtained using the unmasked, partly masked, and fully masked databases.

Supplementary Fig. 5: Effect of database masking on WNV read alignment

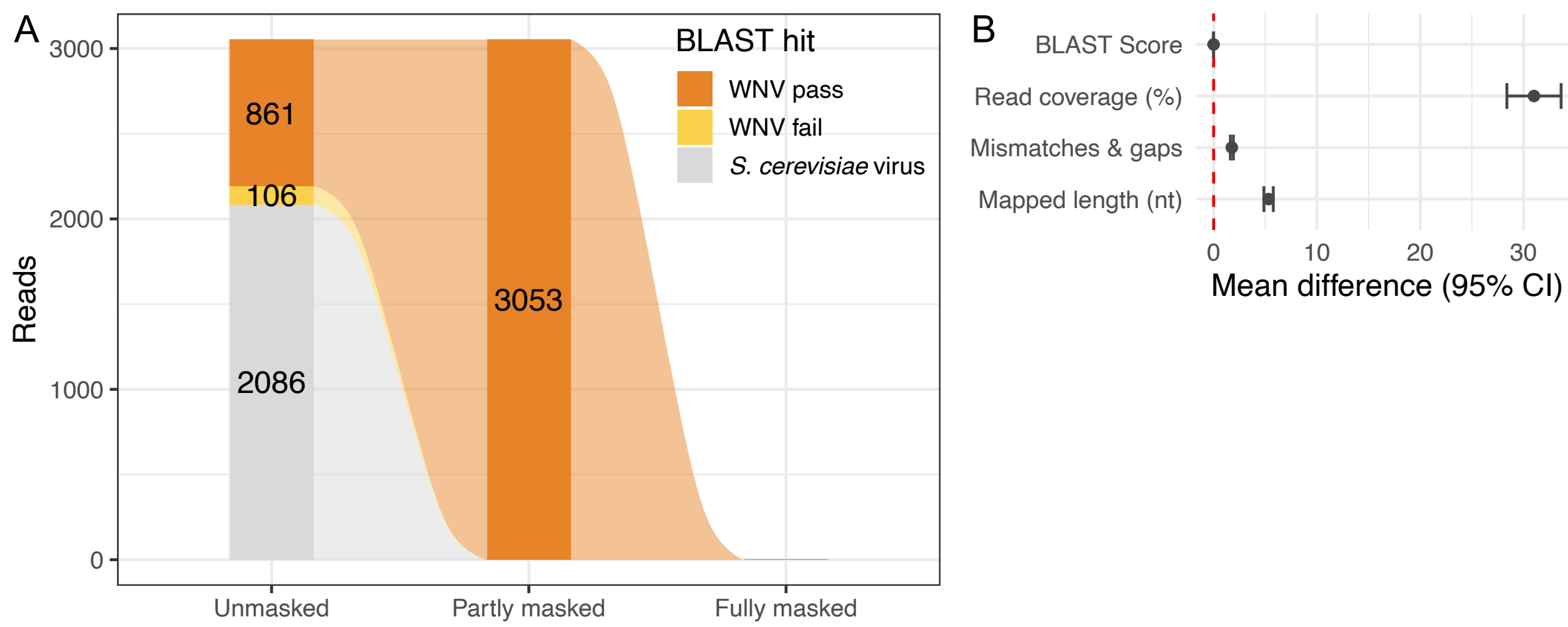

(**A**) The number of reads assigned to West Nile virus (WNV) increased from 861 to 3,053 after the first round of database masking (*Human_masking.py*). Examination of the additional 2,192 reads revealed that over 95% of them (2,086 reads) aligned to *Saccharomyces cerevisiae* virus (MW174761.1) in the unmasked database, while only the remaining 5% (106 reads) aligned to a short WNV region. These 5% of reads matching WNV, however, were never counted as WNV using the unmasked database because, despite sharing a high percent identity with this short WNV region, they did not meet our diagnostic tool (VirMet) assignment criteria (query coverage must be >75%), as the region was shorter than 75% of the read length. Hence, they were discarded. Interestingly, when masking human contaminants from the viral database, the *S. cerevisiae* virus and this short WNV region were masked, preventing the sequencing reads from aligning there. Consequently, when using the partly masked database, all 2,192 reads mapped to another longer region of WNV, allowing the alignments to pass VirMet thresholds (query coverage >75% and percent identity >75%) despite showing a slightly lower percent identity. Subsequent inspection revealed that this second region corresponded to rRNA contamination, which was then masked in the second step as well (*Recurrent_masking.py*), ultimately removing the spurious WNV signal. (**B**) BLAST statistics (score, read coverage, number of mismatches / gaps, and mapped length) of the 106 reads that aligned to WNV using the partly masked database (longer region) as compared to the unmasked database (shorter region). Using the partly masked database (longer WNV region), read coverage was about 30% higher on average than using the unmasked database (shorter WNV region), and the mapped length was about 6 nucleotides longer. However, there were more mismatches and gaps in the middle, decreasing the percentage of identity. Overall, the BLAST score was comparable, which explains why both alignments were considered BLAST “hits”.

Supplementary Fig. 6: Parameter validation on quality control samples

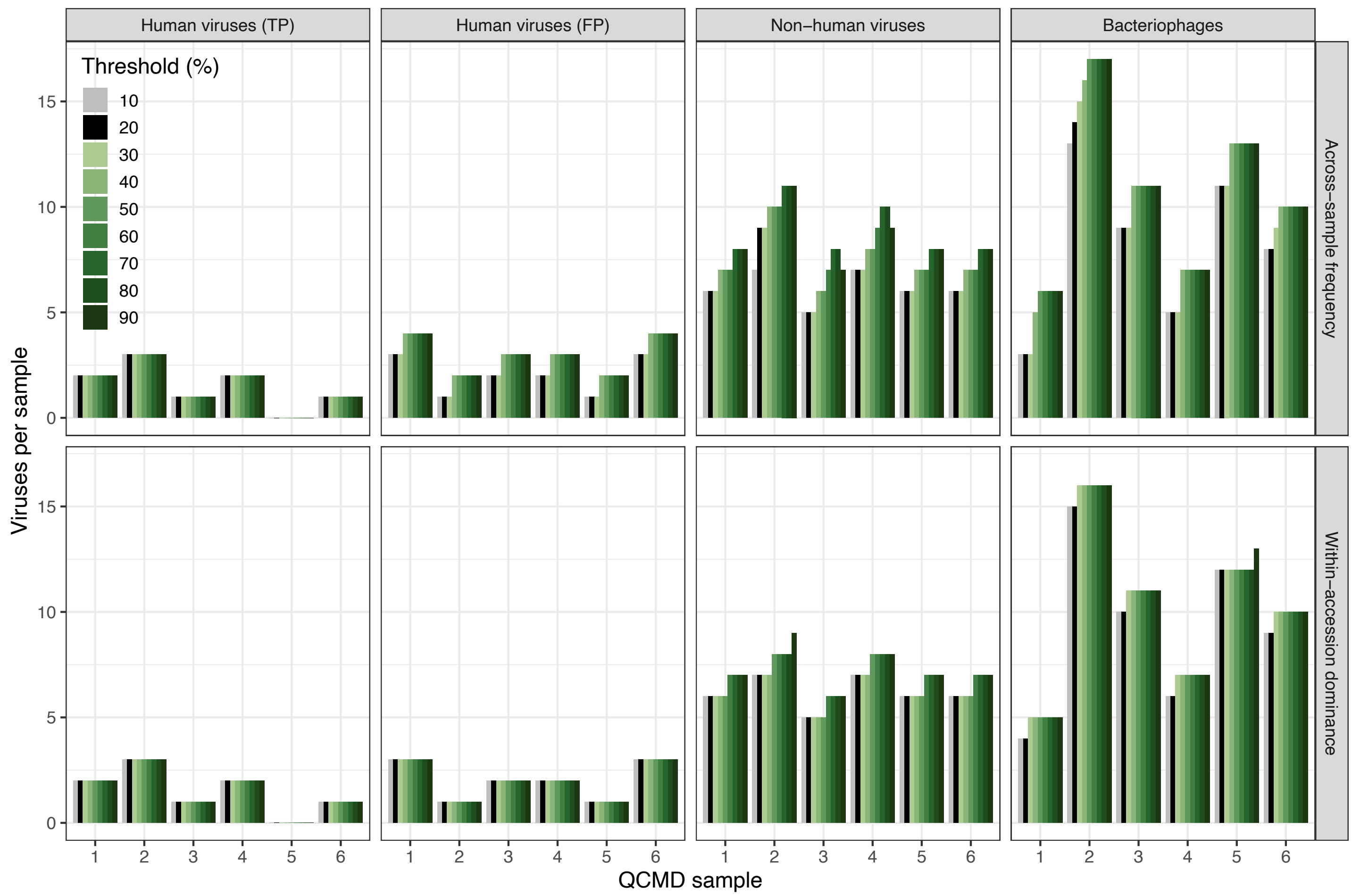

Number of detected viruses across QCMD samples, categorized as human viruses (subdivided into true positives [TP] and false positives [FP]), non-human viruses, and bacteriophages, shown for iterative parameter thresholds for across-sample frequency and within-accession dominance. The black bar (20%) indicates our selected threshold applied to both criteria.
